# Global health training among U.S. residency specialties: a systematic literature review

**DOI:** 10.1080/10872981.2016.1270020

**Published:** 2017-01-12

**Authors:** Duncan K. Hau, Luke R. Smart, Jennifer I. DiPace, Robert N. Peck

**Affiliations:** ^a^ Department of Pediatrics, Weill Cornell Medical College, New York, NY, USA; ^b^ Center for Global Health, Weill Cornell Medical College, New York, NY, USA; ^c^ Department of Medicine, Weill Bugando School of Medicine, Mwanza, Tanzania

**Keywords:** Global health training, residency, post-graduate training, international health elective

## Abstract

**Background:** Interest in global health training during residency is increasing. Global health knowledge is also becoming essential for health-care delivery today. Many U.S. residency programs have been incorporating global health training opportunities for their residents. We performed a systematic literature review to evaluate global health training opportunities and challenges among U.S. residency specialties.

**Methods:** We searched PubMed from its earliest dates until October 2015. Articles included were survey results of U.S. program directors on global health training opportunities, and web-based searches of U.S. residency program websites on global health training opportunities. Data extracted included percentage of residency programs offering global health training within a specialty and challenges encountered.

**Results:** Studies were found for twelve U.S. residency specialties. Of the survey based studies, the specialties with the highest percentage of their residency programs offering global health training were preventive medicine (83%), emergency medicine (74%), and surgery (71%); and the lowest were orthopaedic surgery (26%), obstetrics and gynecology (28%), and plastic surgery (41%). Of the web-based studies, the specialties with the highest percentage of their residency programs offering global health training were emergency medicine (41%), pediatrics (33%), and family medicine (22%); and the lowest were psychiatry (9%), obstetrics and gynecology (17%), and surgery (18%). The most common challenges were lack of funding, lack of international partnerships, lack of supervision, and scheduling.

**Conclusion:** Among U.S. residency specialties, there are wide disparities for global health training. In general, there are few opportunities in psychiatry and surgical residency specialties, and greater opportunities among medical residency specialties. Further emphasis should be made to scale-up opportunities for psychiatry and surgical residency specialties.

## Introduction

The interest in and importance of global health training during residency continues to increase. More than 25% of graduating U.S. medical students have international health experience before starting residency [1]. Many will preferentially rank higher residency programs that offer global health training [2]. This has spurred residency programs to incorporate global health training opportunities for their residents. Between 1995 and 2006 the proportion of U.S. pediatric residency programs offering international health electives doubled from 25% to 52% [3]. Studies have shown residents gain numerous benefits through participation in international health electives including expanded medical knowledge, improved physical examination skills, reduced dependence on laboratory and/or radiologic tests, increased cultural competency, and enhanced professionalism. Additionally, some participants will go on to pursue careers in primary care, obtain a public health degree, or volunteer internationally in the future [4–7].

Knowledge of global diseases has also become essential for health-care delivery today [8]. Population migration and ease of travel have resulted in the globalization of diseases and the importation of emerging infectious diseases such as the Ebola and Zika viruses. Physicians today must be able to diagnose and treat diseases originating in all regions of the world. As more people immigrate around the world, the cultural sensitivity attained through global health education also becomes indispensable.

With the increased interest in global health training and the importance of global health knowledge for physicians, medical educators in the U.S. have tried to capture the proportion of residency programs within a particular specialty that offer global health training. These studies have either surveyed residency program directors or conducted searches of residency program websites. Currently to our knowledge, there is no collective review of these studies. This paper provides a systematic literature review of global health training among U.S. residency specialties. The aim of this literature review is to (1) evaluate which of the U.S. residency specialties are more likely to have global health training opportunities for their residents, and (2) report the challenges frequently encountered with offering global health training.

## Methods

### Data source and search strategies

We conducted a systematic literature review in PubMed for articles related to global health training in U.S. medical and surgical residency specialties. The list of residency specialties was generated from the Accreditation Council for Graduate Medical Education (ACGME) website [9]. The residency specialties included allergy and immunology, anesthesiology, colon and rectal surgery, dermatology, diagnostic radiology, emergency medicine, family medicine, internal medicine, medical genetics, neurological surgery, neurology, nuclear medicine, obstetrics and gynecology, ophthalmology, orthopaedic surgery, otolaryngology, pathology, pediatrics, physical medicine and rehabilitation, plastic surgery, preventive medicine, psychiatry, radiation oncology, surgery, thoracic surgery, and urology. Search terms included combinations and alternatives of the following key terms in association with each ACGME residency specialty: global health, international health, education, curriculum, teaching, elective, and rotation (for the complete search strategy, see Appendix). The search was conducted on October 22nd 2015 and was limited to English language publications with no restrictions on publication date or status.

### Study selection

Two independent reviewers (DH, LS) screened the articles according to *a priori* set of inclusion and exclusion criteria. We included articles that reported survey results of U.S. residency program directors on global health training opportunities within a residency specialty, or reported web-based searches of U.S. residency program websites on global health training opportunities within a residency specialty. We excluded articles that reported survey results of residents or departmental faculty, survey results of fellowship programs or medical schools, not U.S. residency programs, opinion or non-research pieces, non-educational pieces, or not the most recent publication for that residency specialty. Titles and abstracts were screened first. Any remaining articles were then evaluated in full text along with their references to identify additional publications. For residency specialties that had articles published based on surveys and web-based searches, both types of articles were included for the literature review.

### Data collection and synthesis

Data extracted included type of data collection (survey versus web-based search), survey or web-based search year, number of program respondents of survey or number of residency program websites searched, percentage offering global health training within a residency specialty, and challenges in starting or sustaining global health training. If the percentage of challenges were mentioned, this was also extracted.

## Results

**Figure 1.**
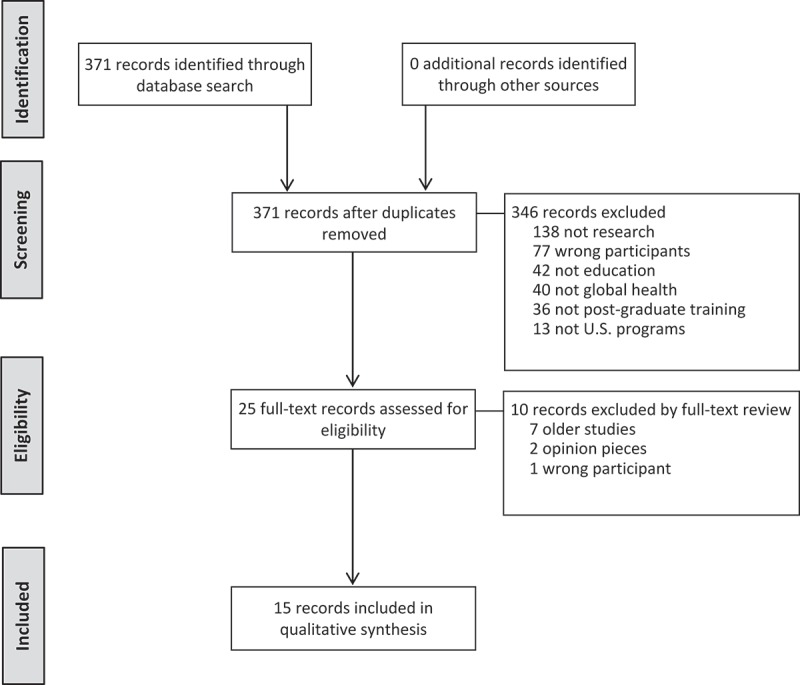
Flow diagram for identification, screening, eligibility, and inclusion of records.

The keyword search yielded 15 articles for this review (Figure 1). Articles were found for the following twelve specialties: emergency medicine, family medicine, internal medicine, neurology, obstetrics and gynecology, ophthalmology, orthopaedic surgery, pediatrics, plastic surgery, preventive medicine, psychiatry, and surgery (Table 1). Articles were not found for the following specialties: allergy and immunology, anesthesiology, colon and rectal surgery, dermatology, diagnostic radiology, medical genetics, neurological surgery, nuclear medicine, otolaryngology, pathology, physical medicine and rehabilitation, radiation oncology, thoracic surgery, and urology.

**Table 1.** Characteristics of the 15 articles describing global health training among 12 training specialties.

Training specialty	Study author and publication date	Data collection	Study year	Response rate	Percentage with global health training	Challenges
Emergency Medicine	Havryliuk *et al*. 2014 [10] Kerry *et al*. 2013 [11]	E-mail Survey Web-based search	2010–11 2011	102 /192* (53%) 155 programs searched	74% (75/102) 41% (64/155)	Lack of funding (42%) Lack of preparation for international work (40%)
Family Medicine	Schultz *et al*. 1998 [12] Kerry *et al*. 2013 [11]	Mail Survey Web-based search	1996 2011	144 /192 (75%) 451 programs searched	54% (73/144) 22% (97/451)	Lack of funding Lack of supporting faculty
Internal Medicine	Kolars *et al*. 2011 [13] Kerry *et al*. 2013 [11]	E-mail Survey Web-based search	2009 2011	279 /366 (76%) 380 programs searched	57% (160/279) 20% (75/380)	Lack of funding Lack of supervision Lack of interest by residents
Neurology	Lyons *et al*. 2014 [14]	E-mail Survey	2012–13	131 /208^┼^ (63%)	50% (65/129)	Lack of funding (86%) Lack of partnerships (55%) Lack of supervision (40%)
Obstetrics and Gynecology	Eichelberger *et al*. 2015 [15] Hung *et al*. 2013 [16]	E-mail Survey Web-based search	2013 2010–11	105 /236 (45%) 243 programs searched	28% (29/105) 17% (41/243)	Lack of funding (80%) Time constraints (71%) Constraints by ACGME (31%) Intensive service requirements Smaller class size
Ophthalmology	Coombs *et al*. 2015 [17]	E-mail Survey	2012–13	59 /116 (51%)	54% (32/59)	Lack of funding (78%) Inadequate resident coverage (67%) Constraints by ACGME (63%) Lack of partnerships (33%)
Orthopaedic Surgery	Shultz *et al*. 2015 [18]	E-mail Survey	Does not mention	73 /154 (47%)	26% (19/73)	Intensive service requirements (74%) Lack of funding (70%) Lack of partnerships (34%)
Pediatrics	Butteris *et al*. 2015 [19] Kerry *et al*. 2013 [11]	Phone, in-person or by E-mail Survey Web-based search	2013–14 2011	198 /199 (99%) 198 programs searched	58% (115/198) 33% (65/198)	Lack of funding Lack of administrative support Lack of partnerships Lack of sharing of curricular and other resources Lack of evaluation tools
Plastic Surgery	Nayar *et al*. 2015 [20]	E-mail Survey	2013	64 /81 (79%)	41% (26/64)	Constraints by RRC (71%) Lack of funding (66%) Salary support (63%)
Preventive Medicine	Bussell *et al*. 2015 [21]	E-mail Survey	2013	23 /42 (55%)	83% (19/23)	Lack of funding (87%) Scheduling (57%) Lack of partnership (35%) Lack of supervision (30%) Liability concerns (26%) Constraints by ACGME (26%)
Psychiatry	Tsai *et al*. 2014 [22]	Web-based search	2010–11	183 programs searched	9% (17/183)	Cultural and/or language Lack of partnerships Lack of funding
Surgery	Knudson *et al*. 2015 [23]	E-mail Survey	Does not mention	48 /253 (19%)	71% (34/48)	Lack of funding Intensive service requirements Cultural differences Gaining support from chair, dean, CMO Meeting the needs of the host Logistics: housing/certification Local issues such as strikes
	Wackerbarth *et al*. 2015 [24]	Web-based search	2014–15	239 programs searched	18% (42/239)	Administrative constraints Lack of funding Accreditation

Abbreviations: ACGME = Accreditation Council for Graduate Medical Education; RRC = Residency Review Committee

* 156 ACGME-accredited & 36 non ACGME-accredited

^┼^ Only U.S. residency programs in a survey of U.S. and Canadian programs

^¶^ Percentages of challenges were not available for all articles

The year of data collection ranged from 1996 to 2015. Two articles did not mention the year of data collection but were published in 2015. Survey results alone were available for five specialties: neurology, ophthalmology, orthopaedic surgery, plastic surgery, and preventive medicine. Web-based search results alone were available for one specialty: psychiatry. Both survey and web-based search results were available for six specialties: emergency medicine, family medicine, internal medicine, obstetrics and gynecology, pediatrics, and surgery. For survey based studies, the percentage of residency programs offering global health training ranged from a low of 26% in orthopaedic surgery, to a high of 83% in preventive medicine. The response rate for surveys ranged widely from 19% of surgical programs reporting to 99% of pediatric programs reporting. The three residency specialties with the highest percentage of their residency programs offering global health training based on survey results were preventive medicine (83%), emergency medicine (74%), and surgery (71%). The three with the lowest were orthopaedic surgery (26%), obstetrics and gynecology (28%), and plastic surgery (41%).

For web-based studies, the percentage of residency programs offering global health training ranged from a low of 9% in psychiatry, to a high of 41% in emergency medicine. The three residency specialties with the highest percentage of their residency programs offering global health training based on web-based results were emergency medicine (41%), pediatrics (33%), and family medicine (22%). The three with the lowest were psychiatry (9%), obstetrics and gynecology (17%), and surgery (18%). For the six residency specialties that had both types of data collection, web-based search results consistently reported a lower proportion of residency specialties offering global health training. This ranged from 11–53% lower than results reported by survey based studies.

The most common challenges included lack of funding, lack of international partnerships, lack of supervision, and scheduling or finding coverage for away residents. Surgical residency specialties reported additional barriers which were intensive service and technical requirements required of training, and lack of approval of resident activities abroad towards graduation.

## Discussion

Wide disparities exist in the availability of global health training opportunities across U.S. residency specialties, ranging from a low of 9% in psychiatry to a high of 83% in preventive medicine. In general, there are fewer global health training opportunities among the surgical residency specialties, and more global health training opportunities among the medical residency specialties. An exception is psychiatry, which has the lowest percentage of all residency specialties found in our literature review. Tsai *et al* speculate that culture and/or language is a major barrier for psychiatry compared to specialties that are more procedural based [22]. The wide disparities in global health training opportunities do not correlate with the global burden of diseases. Both psychiatric and surgical diseases are common globally. Rather, the fewer opportunities for global health training in psychiatry and surgical specialties seems more related to the additional challenges these specialties face compared to medical specialties.

Our literature review found common challenges mentioned across specialties in offering global health training. The most common challenge is lack of funding for residents to partake in an international health elective. Among the neurology residency programs that allow international health electives, most (56%) have no financial support available to participants [14]. Among the internal medicine residency programs that allow international health electives, only 40% report the availability of additional funding for costs like travel and lodging [13]. Furthermore, it can be difficult for residents to fund electives themselves when there is little financial support from their residency programs. Surveys of residents interested in international health electives mention personal financial constraints as a frequent barrier [25]. For residency programs that want to improve funding, they will need to look for potential donors, grants, private philanthropy, and corporate entities with similar interest. For residency programs with persistently scarce funds, global health training could include didactics, journal club, and peer education on global health topics at the home institution rather than an international clinical rotation component.

Lack of international partnerships and lack of supervision are two other common challenges mentioned. Adequate supervision for visiting residents is a critical criterion for establishing an international health elective, since lack of supervision is harmful to both patient care and residents’ global health experiences. When residency programs have faculty aboard, they can provide supervision for the visiting residents. When programs do not have faculty aboard, supervision must be provided by local healthcare providers. There are a limited number of locations where local providers have adequate time and experience to supervise visiting residents. The lack of suitable international partner sites often leads to several residency programs sharing the same site. While there are benefits to this setup, it can create a significant burden on the host institution in terms of oversight of visiting residents and conflict of competing interests between the various institutions.

Scheduling or finding coverage for away residents is another frequent challenge mentioned. Program directors find it difficult to grant call-free elective blocks, especially for programs of smaller size. Residents may need to use their own limited vacation time for their international health electives, which can deter them from going abroad. While there is little consensus on the ideal length of time abroad, residents and program directors might agree that less than six weeks is considered too short, and two or three months is more valuable [8]. Some residency programs may need to use a combination of call-free elective blocks and vacation time to provide residents adequate time abroad.

Surgical residency programs encounter additional barriers in offering global health training. These challenges include intensive service and technical requirements, and lack of approval of resident activities abroad towards graduation requirements. For surgical residents who do not receive credit for clinical activities performed abroad, they must fulfill their graduation requirements in a shorter period of time at the home institution. In recent years, some surgical residency review committees (RRC) have begun to approve international health electives toward program graduation requirements. For example, in 2011 the RRC for general surgery and the American Board of Surgery developed guidelines to allow cases performed abroad to be credited toward program completion [23]. In 2013, the RRC for ophthalmology began allowing credit for international rotations up to one month duration [17]. Since surgical care is multidisciplinary, collaboration with other medical fields like anesthesia, critical care and nursing will be beneficial to allow higher level of surgical care. Also, there has been suggested the creation of a list of core overseas sites for surgical global health electives, therefore allowing pooling together of resources for a greater effect on the host institution and visiting residents’ education [26]. Further collaboration and having core international sites may increase the likelihood of surgical RRC approval and the ability to meet graduation requirements abroad.

Our literature review has several limitations. While our review was able to evaluate twelve residency specialties in regards to global health training opportunities, we did not come across articles for all U.S. medical and surgical residency specialties. The search terms we used could have missed articles describing global health training; however, it can be speculated that some of these specialties being small in size or having intensive service requirements have limited to no opportunities for global health training and therefore have no published studies of this scope. Further medical education studies are needed to capture the available global health training opportunities in these specialties. Another limitation of our review is the use of survey-based studies and web-based studies. Survey-based studies are affected by response bias and response rates. Residency program directors are more likely to respond to global health surveys if their programs offer global health training, which could result in a higher percentage rate reported than actual. Web-based searches rely on information found on residency program websites, which can be incomplete or out of date. Different reported results for the same residency specialty were noted between survey and web-based. The web-based studies consistently reported a lower percentage, ranging from 11–53% below the results of the survey-based studies.

## Conclusion

Among U.S. residency specialties, there are wide disparities in the opportunities for residents to participate in global health training. In general, there are fewer opportunities among psychiatry and surgical residency specialties, and greater opportunities among medical residency specialties. Further effort and exploration should be placed on scaling up global health training opportunities for psychiatry and surgical residency specialties. Neglected mental health illnesses and surgical diseases are a global health crisis, which needs more attention. Expanding global health training among residency specialties will take considerable effort, but trained physicians with global health knowledge will become essential in both the developing and developed world, as mobilization of people and globalization of diseases become more prevalent.

## AppendixSearch Strategy

(“Global health”[mh] OR “global health”[tiab] OR “international health”[tiab] OR “global

surgery”[tiab])

AND

(“Internship and residency”[mh] OR “residency”[tiab] OR “resident”[tiab] OR “allergy and

immunology”[tiab] OR “anesthesiology”[tiab] OR “colon and rectal surgery”[tiab] OR

‘dermatology’[tiab] OR ‘diagnostic radiology’[tiab] OR ‘emergency medicine’[tiab] OR

‘family medicine’[tiab] OR ‘internal medicine’[tiab] OR ‘medical genetics’[tiab] OR

“neurological surgery”[tiab] OR “neurology”[tiab] OR “nuclear medicine”[tiab] OR “obstetrics

and gynecology”[tiab] OR “ophthalmology”[tiab] OR “orthopaedic surgery”[tiab] OR

“otolaryngology”[tiab] OR “pathology”[tiab] OR “pediatrics”[tiab] OR “physical medicine and

rehabilitation”[tiab] OR “plastic surgery”[tiab] OR “preventive medicine”[tiab] OR

‘psychiatry’[tiab] OR ‘radiation oncology’[tiab] OR ‘surgery’[tiab] OR ‘thoracic surgery’[tiab]

OR ‘urology’[tiab])

AND

(‘education’[tiab] OR ‘curriculum’[tiab] OR ‘teaching’[tiab] OR ‘elective’[tiab] OR

‘rotation’[tiab] OR ‘curriculum’[mh])
